# Randomized in situ clinical trial evaluating erosion protection efficacy of a 0.454% stannous fluoride dentifrice

**DOI:** 10.1111/idh.12379

**Published:** 2019-02-19

**Authors:** Nicola X. West, Tao He, Nikki Hellin, Nicholas Claydon, Joon Seong, Emma Macdonald, Svetlana Farrell, Rachelle Eusebio, Aneta Wilberg

**Affiliations:** ^1^ Clinical Trials Unit Bristol Dental School Bristol UK; ^2^ Procter & Gamble Mason Business Center Mason Ohio USA

**Keywords:** clinical trial, dental erosion, dentifrice, oral hygiene, stannous fluoride

## Abstract

**Objectives:**

To assess the protective effects of a 0.454% stabilized stannous fluoride dentifrice and a marketed triclosan dentifrice against enamel erosion in a 10‐day in situ model.

**Methods:**

This was a double‐blind, randomized, 2‐treatment, 4‐period, crossover in situ trial involving healthy adult participants. Participants were randomized to a treatment sequence involving the following products: a highly bioavailable 0.454% stannous fluoride dentifrice (Procter & Gamble) and a marketed dentifrice control containing 0.24% sodium fluoride and 0.3% triclosan (Colgate‐Palmolive). Each study period took place over 10 days. Participants wore an intra‐oral appliance retaining two polished human enamel samples for 6 hours per day. Two times per day they swished with the assigned dentifrice slurry and four times per day they swished with 250 mL of orange juice (25 mL per minute) over a 10‐minute period. Contact profilometry measurements were made for each sample at baseline and day 10 to determine surface change.

**Results:**

Thirty‐six participants were enrolled and 33 completed the study (mean age = 40.5 years). The stannous fluoride dentifrice demonstrated 93.5% less enamel loss than the NaF/triclosan dentifrice (*P* < 0.001) at Day 10, with median enamel loss of 0.097 µm and 1.495 µm, respectively. Both products were well tolerated.

**Conclusion:**

The stannous fluoride dentifrice demonstrated significantly greater erosion protection efficacy relative to the NaF/triclosan dentifrice in this randomized in situ clinical trial.

## 1 INTRODUCTION

Dental erosion is a common problem that has received significant attention in the literature.^1^ Although dental erosion can be found across all age groups, it is of particular concern when identified in very young children due to the development of dietary habits that result in irreversible enamel loss. In a sampling of young adults, Bartlett et  al^2^ reported 29% of participants 15‐35 years old showed symptoms of erosive tooth wear. In a systematic literature review, Salas et  al^3^ reported a 30.4% prevalence of dental erosion in children and adolescents, with high heterogeneity between the studies included in their assessment. Due to the progressive nature of dental erosion, it is not surprising that increases in tooth wear have been positively associated with age^4^, ^5^; the longer we maintain our natural teeth, the more likely we are to experience conditions that are commonly associated with dental erosion.

One of the primary factors associated with dental erosion is lifestyle. Lifestyle trends have changed significantly over the past few decades, such as the dramatic increase in the consumption of acid‐containing drinks, particularly soft drinks, energy drinks and sports drinks, and foods.^6^, ^7^ Multiple studies have confirmed significant increases in the consumption of these products.^6^, ^7^, ^8^, ^9^, ^10^

Over the past several decades, we have gained a much better perspective on the ability of daily hygiene practices, particularly those that include fluoride therapy, to help prevent and even reverse, the caries process.^11^, ^12^, ^13^ Although similar in some aspects, dental erosion is different from caries. Due to the general irreversibility of erosion, many dental professionals are concerned that dental erosion could pose a significant threat to the long‐term health and integrity of tooth structure. As a result, preventive strategies designed to protect exposed tooth surfaces against irreversible damage due to dental erosion are of high interest to the dental community.^1^, ^14^ While some fluoride sources have been shown to provide a limited level of erosion protection, stannous fluoride, a multi‐benefit active used in some toothpastes, has been shown to provide significant anti‐erosion benefits in various types of laboratory studies.^15^, ^16^, ^17^, ^18^ There is a growing body of in situ evidence to support the claim that the use of stabilized stannous fluoride dentifrice is an effective means to help control both the initiation and progression of dental erosion in vivo.^19^, ^20^, ^21^, ^22^, ^23^, ^24^

The study reported here was the first to evaluate a high bioavailable 1100 ppm fluoride stabilized stannous fluoride dentifrice relative to a marketed 1100 ppm fluoride triclosan dentifrice control for erosion protection efficacy using an in situ erosion model with 10‐day treatment periods.

## 2 STUDY POPULATION AND METHODS

### 2.1 Study population

Healthy male and female participants, 18 years of age or older, were recruited from staff at Bristol University and the Bristol Dental School and Hospital from April to June 2016. A sufficient number of participants were recruited to enrol approximately 36 participants in the study. Potential participants attended a screening visit approximately one month before the start of the study.

Prior to receiving any study specific procedures, participants were asked to read a participant information sheet and sign an informed consent form. Qualified participants were required to have no evidence of the following: susceptibility to acid regurgitation; recurrent or regular aphthous ulcers; dental erosion or a previous history of susceptibility to high dental erosion after drinking sports drinks/juices; excessive gingival inflammation; severe periodontal disease; unremovable mouth or tongue jewellery. Participants had to agree to delay elective dentistry, and to refrain from participation in any other product studies. Participants meeting all study entrance criteria were enrolled in the study and scheduled to return for treatment.

### 2.2 Ethical considerations

This human in situ clinical study was granted ethical approval by the UK National Research Ethics Service (NRES Committee South West—Central Bristol, REC Ref: 15/SW/0266), and the study was designed and managed in compliance with the principles of Good Clinical Practice. The study was conducted at the Clinical Trials Unit of Bristol Dental School and Hospital, Lower Maudlin Street, Bristol, UK.

### 2.3 Randomization

Participants presented for four study periods and were randomized to one of four treatment sequences by a computer‐generated sequence (AABB, BBAA, ABBA or BAAB, where the letters correspond to the two study treatments) that determined the participant's use of one of the two commercially available test dentifrices each period: (a) a highly bioavailable 0.454% stannous fluoride dentifrice (Crest^®^ Pro‐Health^™^ Advanced Gum Protection, Procter & Gamble, Cincinnati, OH, USA; 1100 ppm fluoride); or (b) a marketed dentifrice control containing 0.24% sodium fluoride and 0.3% triclosan (Colgate^®^ Total^®^, Colgate‐Palmolive, New York, NY, USA; 1100 ppm fluoride).

### 2.4 In situ study design

This was a single centre, double‐blind, randomized, two‐treatment, four‐period crossover study that was a variation of the previously published method of Hooper et  al^24^. During the screening visit prior to the start of study treatments, recruited participants were provided with a non‐treatment 0.32% NaF (1450 ppm fluoride) marketed dentifrice (Crest^®^ Decay Protection dentifrice, Procter & Gamble) and manual toothbrushes (Oral‐B^®^35 manual toothbrush, Procter & Gamble) to use at home, both prior to and during the course of the study. Participants were required to use these two products in place of their normal oral care products, twice per day (morning and evening) for the duration of the study, including treatment days and weekends.

**Figure 1:**
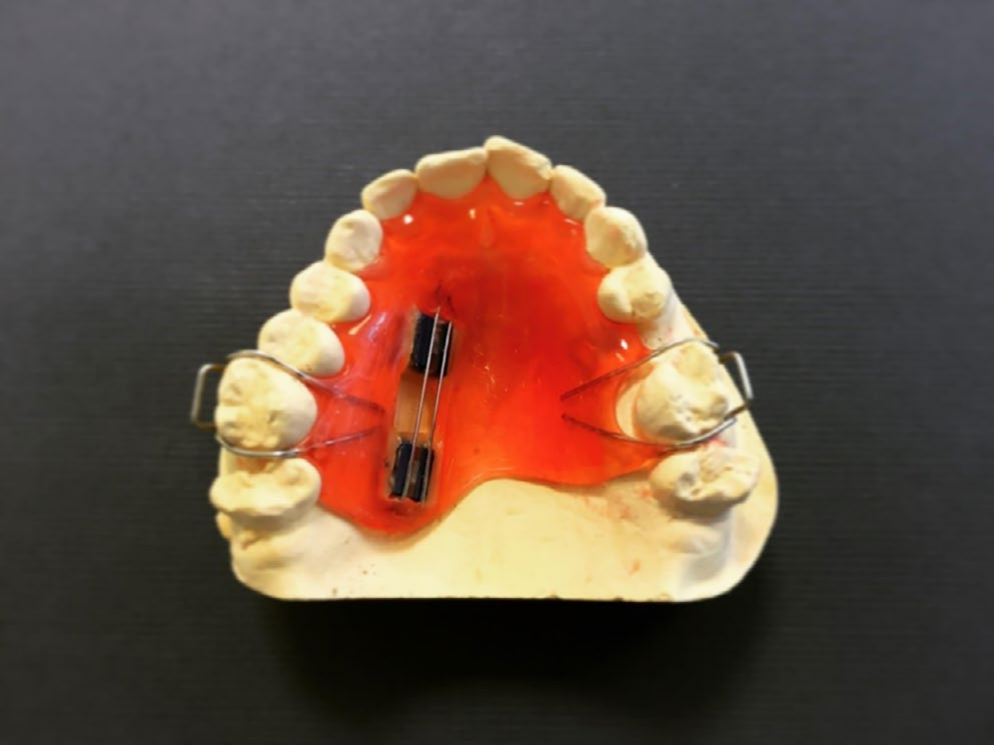
Intraoral appliance fitted with two enamel samples

**Figure 2:**
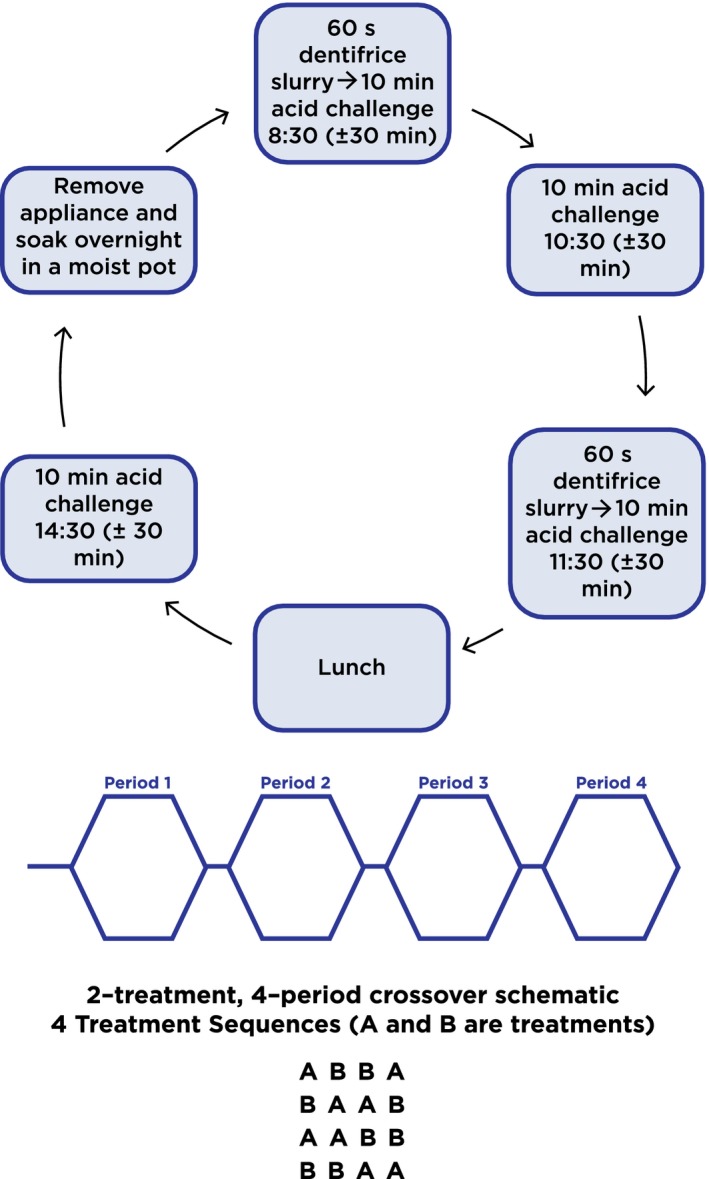
Study design with treatment and acid challenge schedule

Study participants were fitted with an upper palatal intraoral appliance (Figure 1). Each appliance was fitted with two enamel samples derived from unerupted third molars from a Tooth Tissue Bank in the UK. Upon arrival at the clinical trials unit on each study treatment day, participants collected their intraoral appliance with the fitted enamel specimens and placed it in their mouth; wearing it for approximately 6 hours on each of the 10 treatment days. While wearing the appliance, participants swished twice each day for 60 seconds with their assigned dentifrice slurry at the clinical site. The slurry was prepared by the clinical site personnel as follows: Three (3.0) grams of dentifrice were mixed with 10 mL of water, with participants being unaware of the product identity of their assigned dentifrice slurry. The erosive challenge occurred four times on each day of the treatment phase of the study, with the intraoral appliance in place. (Figure 2) Participants sipped 25 mL of orange juice (Sainsbury's Supermarkets Ltd, London, UK) over a timed minute, swished it around their mouth and then spat it out. This procedure was repeated 10 times so that the enamel samples were exposed to a total of 250 mL of orange juice over each 10‐minute period of erosive challenge. Each study period was comprised of 10 treatment days, with treatments being done only on weekdays (Monday‐Friday) over a span of about 2 weeks. To ensure blinding, the investigator and personnel performing and recording the surface profilometry assessments had no access to the product dispensing room during treatments.

### 2.5 Preparation and analysis of enamel samples

Prior to the start of the study, profilometry measurements were made on each sample using a calibrated contact surface profilometer (Mitutoyo (UK) Ltd, Andover, Hampshire, UK) to establish a baseline surface profile for each enamel specimen. At the end of the treatment phase of the study, each enamel sample was again measured using the same profilometry method, and tissue loss was calculated. Fresh samples of human enamel were fitted into the intra‐oral appliance at the beginning of each of the four treatment periods. A detailed description of procedures related to the acquisition, sterilization, preparation and analysis of human enamel test specimens has been described previously.^24^

### 2.6 Determination of sample size

Using 36 participants, at least 80% power to detect a two‐sided 5% significant difference between the treatment dentifrices would be achieved provided the natural log scale effect size (mean difference divided by the error standard deviation) was 0.70 or greater for this crossover design.

### 2.7 Statistical methods

The primary measure of efficacy in the in situ trial was the amount of dental erosion that had occurred, measured by profilometry, after 10 days of treatment. The average of four erosion measurements was calculated from each of the two enamel specimens for each study participant at each visit. Since the Day 10 enamel loss distribution is right‐skewed, the data were transformed using the natural log function before performing statistical analysis. A general linear mixed model was used to compare treatments with a statistical model that included period and treatment as fixed effect, participants as a random effect and baseline as covariate. The carry‐over effect was not statistically significant (*P* > 0.31) and excluded from the model. From the statistical model, estimated means on the natural log scale were back‐transformed by using the exponential function (*e* ^mean^) to obtain the estimated median or 50th percentiles on the original scale (µm), along with the associated standard errors and/or 95% confidence intervals (CI). Statistical comparisons were two‐sided at a 5% significance level.

The null hypothesis tested at Day 10 in this human in situ clinical study was that the mean dental erosion was equal between the two treatment dentifrices, and the alternative hypothesis was that the mean dental erosion was not equal between the two treatment dentifrices.

## 3 RESULTS

In this randomized in situ clinical evaluation, 36 participants ranging in age from 20‐60 (mean age 40.5 years) were enrolled and 35 were randomized to treatment. Twenty‐four (69%) participants were female. (Table 1). Thirty‐three (33) participants completed the study, and all study data were deemed evaluable. Three participants dropped due to voluntary withdrawal from the study.

**Table 1:** Study demographics

Demographic	Statistic or category	Value
Age (y)	Mean (SD)	40.5 (13.48)
	Min.–Max.	20‐60
Ethnicity^a^	Asian Indian	2 (6%)
	Asian Oriental	1 (3%)
	Caucasian	32 (91%)
Gender^a^	Female	24 (69%)
	Male	11 (31%)

a The number and percent of subjects in each category.

**Figure 3:**
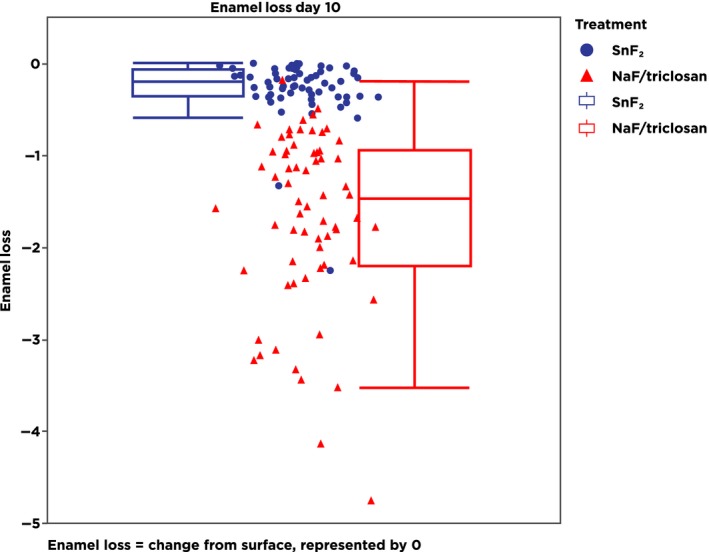
Box plot of data showing individual and averaged enamel loss data points for each of the two test dentifrices

At Day 10, the stabilized stannous fluoride dentifrice demonstrated 93.5% less enamel loss (*P* < 0.0001) vs the NaF/triclosan multi‐benefit dentifrice, with estimated enamel loss medians (CI) of 0.097 µm (0.074, 0.127) for the stabilized stannous fluoride dentifrice and 1.495 µm (1.157, 1.931) for the NaF/triclosan dentifrice (Table 2). Distribution Box Plots of enamel loss by treatment (Figure 3) verify distinct differences in performance between the two test dentifrices at Day 10.

**Table 2:** Enamel loss (µm) treatment comparison at Day 10

Treatment	Original scale in µm estimated median (95% CI)	Natural Log Scale Mean (SE)	% Less erosion vs NaF/triclosan (*P*‐value)^a^
Stannous fluoride	0.097 (0.074, 0.127)	−2.336 (0.136)	93.5% (*P* < 0.0001)
NaF/triclosan	1.495 (1.157, 1.931)	0.401 (0.128)	

a Calculated from estimated medians in μm as 100% (NaF/triclosan—Stannous fluoride divided by NaF/triclosan).

Both dentifrices were well tolerated. No significant adverse events were reported.

## 4 DISCUSSION

This study, using 10‐day treatment periods, demonstrated a significant erosion protection benefit of 93.5% for the multi‐benefit, 1100 ppm fluoride, high bioavailable stabilized stannous fluoride dentifrice compared to the triclosan‐containing dentifrice formulated with 1100 ppm fluoride as NaF. These results are consistent with other in situ clinical studies in the literature that have similarly demonstrated enhanced erosion protection, ranging from 27% to 94%, delivered from stabilized stannous fluoride dentifrices compared to other dentifrices that are not formulated with this ingredient.^19^, ^20^, ^21^, ^22^, ^23^, ^24^ For example, a similar study by West et  al^19^ found 68% greater erosion protection for a 0.454% stannous fluoride plus 0.077% sodium fluoride (1450 ppm fluoride) dentifrice marketed in the United Kingdom compared to a 1450 ppm fluoride NaF/triclosan dentifrice after 15 days of treatment and a 67% benefit after 10 treatment days. These study results are also consistent with recommendations from professional groups and organizations.^25^, ^26^ A recent consensus report by the European Federation of Conservative Dentistry noted that dentifrices containing stannous fluoride have the potential for slowing erosive tooth wear, while data for other products is limited.^26^

**Figure 4:**
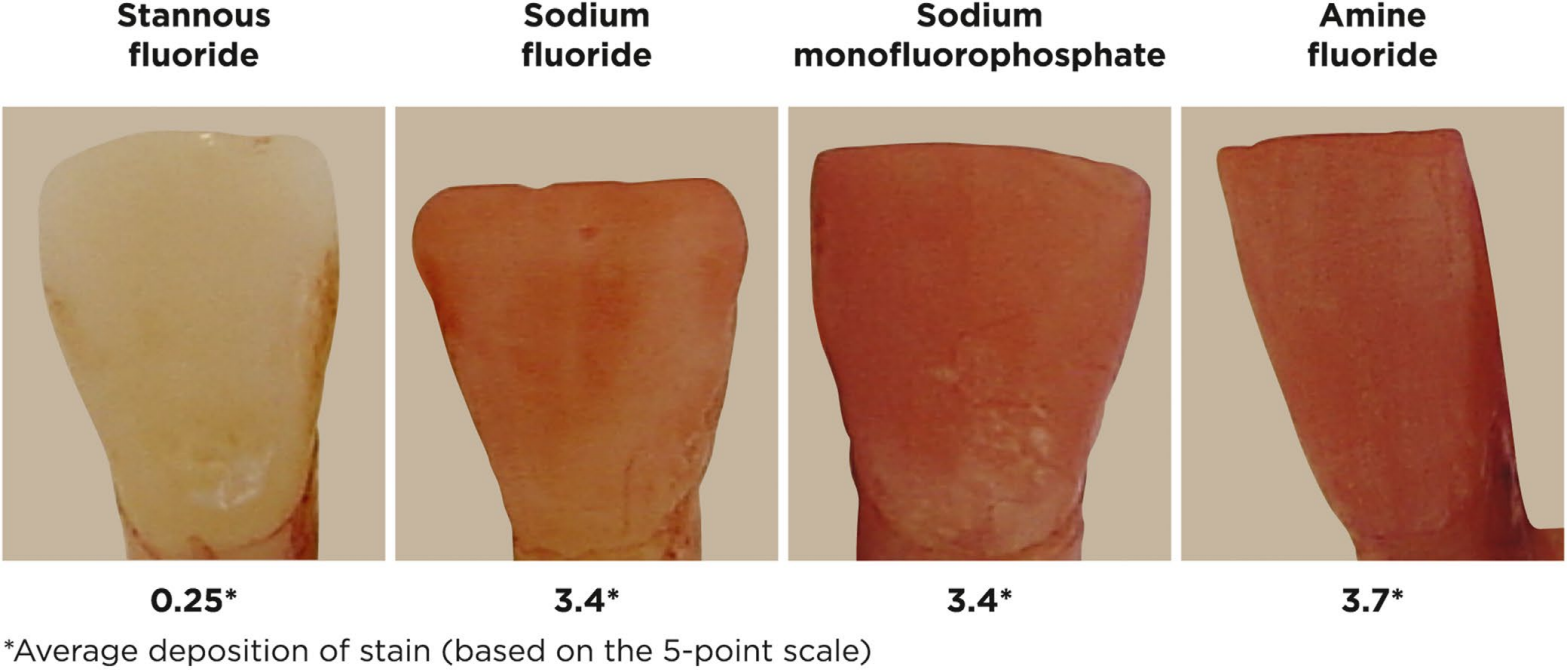
Dye deposition on acid etched enamel samples following treatment with toothpaste/saliva slurry. Samples were treated with a toothpaste/saliva slurry (1100 ppm stannous fluoride, 1100 ppm sodium fluoride, 1000 ppm sodium monofluorophosphate or 1400 ppm amine fluoride) and exposed to 2% alizarin Red‐S following rinsing. Dye deposition was evaluated using a 5‐point scale; 0 indicates no dye deposition and 4 indicates complete dye coverage. A low score signifies the presence of a barrier layer preventing dye deposition^28^

Although both dentifrices included in the current study are marketed as being “multi‐benefit” products, protection against dental erosion is determined, in part, by the ability of a dentifrice to deposit a protective, acid‐resistant barrier layer onto the surface of treated teeth. Stabilized stannous fluoride is capable of depositing such a layer. Mechanism of action studies found that stannous fluoride provides erosion benefits through the deposition of a transparent, protective barrier layer onto the enamel surface that provides enhanced resistance against erosive acid challenges.^16^, ^17^, ^27^ Figure 4 shows images of etched enamel slabs treated with stannous fluoride, sodium fluoride, sodium monofluorophosphate or amine fluoride slurries and stained with 2% alizarin Red‐S.^28^ The slab treated with stannous fluoride showed little evidence of staining with the dye, indicating that it had formed a surface layer that protected against dye deposition. Stannous fluoride is able to incorporate onto both smooth and pellicle‐coated surfaces. The barrier layer that forms on pellicle‐coated enamel, likely composed of metal‐rich precipitates such as Sn_3_F_3_PO_4_, Ca(SnF_3_)_2_ or SnOHPO_4_,^29^, ^30^ increases with continued use and is retained on treated tooth surfaces for extended periods of time, strengthening the enamel surface against subsequent acid attacks.^16^, ^27^ Early stannous fluoride formulations were associated with the potential for surface stain, but contemporary formulas contain stain‐mitigating ingredients, such as the sodium hexametaphosphate in this formula, that can actually provide extrinsic whitening.^31^, ^32^

One might argue the erosion protection benefits of oral care products should be demonstrated in long‐term human erosion clinical studies; however, given the slow progression of the condition, the general irreversibility of dental erosion and ethical considerations, such studies are impractical. Properly controlled in situ studies provide a reasonable clinical alternative for assessing relative erosion protection performance in relatively short periods of time, while minimizing the risk to participants’ natural tooth surfaces.

While dental erosion follows a path that is, in some ways, similar to the demineralization aspects of caries (ie, tooth mineral is lost due to an acid challenge), dental erosion is different; it primarily occurs as a loss of surface, rather than subsurface mineral.^1^ This key factor makes it far more difficult to restore lost mineral through the remineralization process. Although remineralization via fluoride therapy works well in areas of subsurface demineralization, physical challenges to surface minerals, even after fluoride exposure, may be too much for the softened mineral to withstand the level of abrasion that occurs in the mouth on an almost constant basis. Even the tongue can present an almost constant abrasive factor, as it comes into contact with exposed tooth surfaces on a routine basis.^33^, ^34^

The goal of any product intended to help prevent dental erosion is to maintain the integrity of the sound enamel surface. The current model is designed with this in mind, as it assesses the ability of test products to protect sound enamel against erosive softening of the surface mineral that can lead to erosive tissue loss. The erosive challenges, two of which occurred shortly after dentifrice treatment and two of which occurred a few hours post‐treatment, were intended to reflect different time intervals in which erosive challenges might occur post‐brushing over the course of a participant's routine day. While these time intervals might not be exactly the same as those that might occur on a daily basis, the schedule provides a standardized challenge for each treatment group to maximize similarities between treatments and minimize variances. Focusing on prevention, the model does not incorporate direct, physical brushing of the enamel specimens. Physical abrasion is only an issue after the surface has been erosively softened. In vivo, the fluoride‐rich outer layer of enamel, along with the natural pellicle, provides protection against both erosive and abrasive challenges; however, with sufficient challenge, this protective layer is overwhelmed and erosive tissue loss can progress. Treatment of the specimens with slurries, rather than direct brushing, helps ensure that the model remains focused on the preventive aspect of the treatment. By treating all specimens in the same manner, the model measures the relative abilities of the various test products to protect the treated enamel against the erosive acid insult. The ability of stannous fluoride to deposit an acid‐resistant barrier layer onto treated tooth surfaces confirms that this protected mineral was not lost as a result of acid softening, erosive processes. The preventive benefits of stannous fluoride therapy are clearly evident in the current study, with the differences between the two test products being highly significant.

## 5 CONCLUSION

The stannous fluoride dentifrice included in this randomized in situ clinical trial demonstrated significantly greater erosion protection efficacy relative to the NaF/triclosan dentifrice. The use of a clinically proven stannous fluoride dentifrice is both a convenient and economical way to incorporate a preventive strategy for dental erosion into patients’ daily oral hygiene regimen.

## 6 CLINICAL RELEVANCE

### 6.1 Scientific rationale for study

Dental erosion is a growing global oral care issue, and stabilized stannous fluoride dentifrices have been consistently proven to help control both the initiation and progression of this condition better than other tested products.

### 6.2 Principal findings

This in situ clinical study demonstrated greater erosion protection potential of a stabilized stannous fluoride dentifrice compared to a NaF/triclosan dentifrice.

### 6.3 Practical implications

Stabilized stannous fluoride dentifrices should be strongly recommended as part of a comprehensive daily oral hygiene plan, especially for those patients at risk of developing dental erosion.

## STUDY REGISTRATION

This study was registered post hoc: ISRCTN55245733.

## CONFLICT OF INTEREST

Professor West, Ms Hellin, Dr Claydon, Dr Seong and Dr Macdonald are all full‐time employees of the University of Bristol Dental School and Hospital, Bristol, UK. Dr He, Dr Farrell, Ms Eusebio and Ms Wilberg are all full‐time employees of The Procter & Gamble Company, Mason, OH, USA.
